# Transcript Complexity and New Insights of Restorer Line in CMS-D8 Cotton Through Full-Length Transcriptomic Analysis

**DOI:** 10.3389/fpls.2022.930131

**Published:** 2022-06-21

**Authors:** Juanjuan Feng, Yongqi Li, Jinfa Zhang, Meng Zhang, Xuexian Zhang, Kashif Shahzad, Liping Guo, Tingxiang Qi, Huini Tang, Hailin Wang, Xiuqin Qiao, Zhongxu Lin, Chaozhu Xing, Jianyong Wu

**Affiliations:** ^1^State Key Laboratory of Cotton Biology, Institute of Cotton Research of Chinese Academy of Agricultural Sciences, Anyang, China; ^2^National Key Laboratory of Crop Genetic Improvement, College of Plant Science and Technology, Huazhong Agricultural University, Wuhan, China; ^3^Department of Plant and Environmental Sciences, New Mexico State University, Las Cruces, NM, United States

**Keywords:** CMS-D8, restorer gene *Rf_2_*, full-length transcript, novel isoforms, fusion transcript

## Abstract

Hybrid utilization has proficiently increased crop production worldwide. The cytoplasmic male sterility (CMS) system has emerged as an efficient tool for commercial hybrid cotton seed production. The restorer line with dominant *Rf_2_* gene can restore the fertility of the CMS-D8 sterile line. However, the molecular mechanism of fertility restoration remains unclear in CMS-D8 cotton that limits wider utilization of three-line hybrid breeding. In our study, the Pacific Biosciences (PacBio) Iso-Seq technology was applied to understand fertility restoration mechanism of CMS-D8 cotton. In total, 228,106 full-length non-chimeric transcriptome sequences were obtained from anthers of developing flowering buds. The analysis results identified 3,174 novel isoforms, 2,597 novel gene loci, 652 long non-coding RNAs predicted from novel isoforms, 7,234 alternative splicing events, 114 fusion transcripts, and 1,667 genes with alternative polyadenylation. Specially, two novel genes associated with restoration function, *Ghir_D05.742.1* and *m64033_190821_201011/21103726/ccs* were identified and showed significant higher levels of expression in restorer line than sterile and maintainer lines. Our comparative full-length transcriptome analysis provides new insights into the molecular function of *Rf_2_* fertility restorer gene. The results of this study offer a platform for fertility restoration candidate gene discovery in CMS-D8 cotton.

## Introduction

Cotton is prime natural fiber crop widely cultivated worldwide in tropics and subtropics regions. Similar to other crops, the climate change and emergence of new diseases significantly reduce cotton yield and quality. Hybrid breeding is an important measure to reduce recent decline of cotton production (Shahzad et al., 2020). In commercial hybrid seed production, cytoplasmic male sterility (CMS) system is an efficient tool to minimize seed production cost. The CMS is maternally inherited phenomenon, prevents pollen fertility by mitochondrial dysfunction, and facilitates utilization of hybrid vigor (Zhang et al., 2020). The CMS phenomenon exists in more than 150 plants. It has already been exploited in hybrid breeding of many crops such as maize (Laughnan and Gabay-Laughnan, 1983; Jaqueth et al., 2019), rice (Akagi et al., 1994; Chang et al., 2016), pepper (Ma et al., 2013), and sorghum (Tang et al., 1996). In cotton, there are various types of CMS lines, including *G. harknessii* (D_2-2_) cytoplasmic male sterile (CMS-D2) lines (Meyer, 1975; Weaver and Weaver, 1977), *G. trilobum* (D8) cytoplasmic male sterile (CMS-D8) lines (Stewart et al., 1992), 6001A line derived from the wide crossing of *G. thurberi* and *G. hirsutum* (Gao et al., 2021), and upland cotton cytoplasmic male sterile lines (104-7A, Xiangyuan A, Jin A; Zhang, 2005). Within plant CMS systems, pollen sterility can be rescued though regulatory mechanism of different nuclear fertility restorer (*Rfs*) genes (Hanson, 1991; Linke and Börner, 2005; Wu et al., 2011). Different sterile lines have corresponding restorer lines in cotton. The restorer of CMS-D2 contains the *Rf1* gene (Weaver and Weaver, 1977), the restorer of CMS-D8 contains *Rf_2_* (Stewart et al., 1992), and fertility of 6001A sterile line can be restored by the *D05_PPR*-clusters existed in restorer line 7R13 (Gao et al., 2021). Both *Rf_1_* and *Rf_2_* were found to be located on the same chromosome D05 within a genetic distance of 0.9 cM (Zhang and Stewart, 2001b). However, the restorer gene *Rf_1_* of CMS-D2 has the ability to restore fertility of both CMS-D2 and CMS-D8 sterile lines, while fertility of CMS-D2 sterile lines could only be restored by *Rf_1_*. Although there have been several reports on the transcriptome of the cotton CMS system (Suzuki et al., 2013; Wu et al., 2017; Yang et al., 2018; Li et al., 2021). However, no study has reported the novel isoforms and novel gene loci of CMS-D8 restorer line compared with *G. hirsutum* TM-1.

High throughput sequencing technology has recently been made substantial progress to detect complete transcripts, novel genes, isoforms, alternative splicing (AS), open reading frames, and long non-coding RNAs (lncRNAs). Specifically, single-molecule real-time (SMRT) sequencing by PacBio emerged as unique platform to construct full-length transcripts (Roberts et al., 2013). With progress in sequencing technology and bioinformatics, SMRT sequencing was mostly employed in various research projects of corn (Guo et al., 2021), cotton (Li et al., 2021), rice (Schaarschmidt et al., 2020), and clover (Chao et al., 2018). The SMRT sequencing proved more appropriate datasets than short read sequenced technology. It can directly provide all statistics of sequenced RNA without assembly, reads gaps, and high errors (Au et al., 2013; Tilgner et al., 2014; Treutlein et al., 2014; Gordon et al., 2015), this methodology efficiently identified different isoforms of each gene, AS, fusion transcripts, and often improve the accuracy of existing gene models. Furthermore, alignment of different isoforms to the reference genome can effectively identify the modes of alternative splicing of genes. In this way, SMRT improve the accuracy of long isoforms alignment (Kraft and Kurth, 2020). Hence, application of SMRT sequencing can serve as a platform to discover fertility restoration mechanism in CMS-D8 cotton.

For a particular interest, this study performed integrated Iso-seq and RNA-Seq analysis by using mixed anthers of CMS-D8 cotton. Our results characterized full-length transcript differences between restorer, sterile, and the maintainer lines. Further data analysis revealed potential isoforms, alternative splicing events, and fusion transcripts in three lines hybrid cotton. In particular, this study identified 39 novel isoforms that specifically expressed in restorer line. The qRT-PCR analysis stated that *Ghir_D05.742.1* and unmapped *m64033_190821_201011/21103726/ccs* had shown significant higher expression in restorer lines as compared with sterile and the maintainer lines. Our results provide valuable insights into molecular function of fertility restoration in three lines hybrid cotton. These results will offer an important platform to identify the *Rf_2_* restorer genes in CMS-D8 cotton.

## Materials and Methods

### Plant Materials, RNA Extraction, and Illumina RNA-Seq Library Construction

The plant material used in this study contained sterile, maintainer and restorer lines of CMS-D8 system. The sterile line (A) has D8 cytoplasm and contained homozygous recessive fertility restorer alleles (*rf_2_rf_2_*), whereas the maintainer line (B) is fertile with upland cotton (AD1) and has homozygous recessive fertility restorer alleles (*rf_2_rf_2_*). The restorer line (R) is fertile with D8 cytoplasm, and homozygous dominant fertility restorer alleles (*Rf_2_Rf_2_*) to recover fertile anther in cotton. The anther from flower bud samples with length of 1–2, 3, and 4 mm were harvested from 100 plants of each line, with three biological replicates. All harvested anther samples were utilized to prepare composite anther sample, snap-frozen in liquid nitrogen, and stored at −80°C before further use. Total RNA was isolated using the Sigma Spectrum Plant Total RNA kit (Sigma-Aldrich, United States) according to the manufacturer’s protocol. RNA concentration, purity, and integrity were monitored by NanoDrop, agarose gel electrophoresis and Agilent 2100, respectively. Poly (A) mRNA was isolated by poly-T oligo attached magnetic beads (Invitrogen). Following fragmentation, the cleaved RNA fragments were reverse-transcribed into a cDNA library following treatment with the TruseqTM RNA sample prep Kit (Illumina, San Diego, USA). After assessing the library quality, PE 2 × 150 sequencing was performed on an Illumina-Hiseq at the Majorbio (Shanghai, China) following the vendor’s recommended protocol.

### PacBio Sequencing and Data Analysis

Equimolar rations of the anthers total RNA from R line of 1–2, 3, and 4-mm buds in length were combined together. The full-length cDNA for library was synthesized from 1 μg of purified polyA (+) RNA with SMARTer^™^ PCR cDNA Synthesis kit (Clontech). Then, Iso-seq libraries were constructed according to the online available protocol at https://www.pacb.com/wp-content/uploads/Procedure-Checklist-Iso-Seq-Template-Preparation-for-Sequel-Systems.pdf. Once library was prepared and quantified, each SMRT cells were sequenced on the PacBio sequel platform with P6-C4 reagent. PacBio raw data was first handled using the workflow of the PacBio SMRT Analysis software suite.^1^ Briefly, raw polymerase reads were filtered and trimmed to generate the subreads and read of inserts (ROIs) using the RS_Subreads protocol, requiring a minimum polymerase read length of 50 bp, a minimum polymerase read quality of 0.75, a minimum subread length of 50 bp, and a minimum of one full pass. Full-length and non-chimeric (FLNC) reads were regarded as those containing a 5′ adapter, 3′ adapter, and poly (A) tail in the expected arrangement with no additional copies of the adapter sequence within the ROI. Error correction of FLNC reads was performed with the highly quality Illumina short reads using Proovread version 2.12 with the default parameters. The quality of Illumina short reads was examined using FastQC (v0.11.5).^2^ Sequencing adaptors and low-quality bases in short reads were trimmed before the error correction of FLNC reads. FLNC reads before and after error correction were, respectively, mapped to the IWGSC RefSeq v1.0 using GMAP (version 2016-09-14).^3^

### Identification of Gene Loci and Isoforms

According to the read-genome alignments, FLNC reads with the same splicing junctions were collapsed into one isoform. The redundant transcripts were removed through two different ways. First, if all the splicing sites of the same loci transcripts were identical, they were considered one isoform. Secondly, if one isoform was degraded at the 5′ terminal region but the remaining region was consistent with other isoforms, it should be cleaned out. This supporting evidence was examined for the identification of isoforms. The resulted isoforms were retained which was supported with at least two FLNC reads or one FLNC read with percentages of identity higher than 99%, or all junction sites were fully supported by Illumina reads or annotations of the IWGSC RefSeq v1.0. Isoforms that overlapped by at least 20% of their length on the same strand were considered to be from the same gene locus. All detected gene loci and isoforms were matched with the reference annotation to categorize known genes and isoforms as well as novel genes and isoforms. A sequenced gene was regarded to be a novel gene by satisfying any of the following criteria. (i) There is no overlap or an overlap of <20% (ii) The annotated gene has overlap > 20% but the gene direction is inconsistent. In addition, if the sequenced isoform confined one or more new splicing sites, if both sequenced isoform and annotated isoform were not single-exon, the isoform was named as a novel isoform.

### Functional Annotation of the Full-Length Transcriptome

The novel isoforms annotations were retrieved from NR, KOG (Karyotic Ortholog Groups), KO, and Swiss-Prot databases (A manually annotated and reviewed protein sequence database) with Diamond software (Gasteiger et al., 2001; Buchfink et al., 2015). KEGG (Kyoto Encyclopedia of Genes and Genome) pathways were searched using KOBAS v2.0 (Xie et al., 2011). GO (Gene Ontology) annotations were obtained by running BLASTX v2.2.26 and BLAST2GO v2.3.5 software (Conesa et al., 2005).

### LncRNA and ORF Identification

Newly identified isoforms with length ≥ 200 nt were first searched against NCBI’s NR database using BLASTX with default parameters. The isoforms that had BLAST hits with 1E-5 were filtered out, and the remaining isoforms were further evaluated by CPAT v1.2.2.^4^ The transDecoder software was applied to detect potential coding sequences and to predict ORFs.^5^ The length of ORFs predicted by TransDecoder.LongOrfs was at least 100 amino acids by default. To increase the sensitivity of ORFs, potential ORF translated proteins were aligned to the Swiss-Prot database with BlastP for homologous protein identification. Protein domain identification was acquired from the Pfam database using Hmmscan (Eddy, 2009; Finn et al., 2014). Subsequently, TransDecoder. Predict was used to filter all predicted ORFs based on the above results, and retained ORFs that had homology to the Swiss-Prot database or with the same domain.

### Identification of AS and Alternative Polyadenylation

AS events were classified and characterized by comparing different isoforms of the same gene loci using Asprofile (Florea et al., 2013). The alternative polyadenylation (APA) sites for each gene locus were detected using TAPIS (Abdel-Ghany et al., 2016). The number of APA for each gene locus as well as the number of transcripts supporting an APA was provided as data files.

### Fusion Transcript Identification

Fusion transcripts were identified by parsing mapped data using the iso-seq fusion transcripts detection software self-developed by Frasergen Inc. (Wuhan, China). A FLNC was considered as a candidate fusion transcript when all of the following criteria were satisfied:

1. A FLNC must be map to 2 or more annotated genes that are at least 10 Kb apart.
2. Alignment to each gene must have >10% FLNC coverage.
3. The total combined FLNC coverage from all alignments must be >99%.
4. Supported by a certain amount of PE reads across the fusion junction.

### Differential Expression Analysis

Illumina RNA-seq data of equally mixed anthers of 1–2-, 3-, and 4-mm flower buds, respectively, A, B, and R lines. The programs TopHat and Cufflinks were used to blast the sequenced reads against the reference genome of *G. hirsutum*. The analyses of differential genes and transcript expression can evaluate the abundance of gene expression and also reveals new genes that have not been previously annotated in reference genomes. The Fragments Per Kilobase of exon per Million fragments mapped (FPKM) method was used to calculate the abundance of gene expression. DESeq was used to analyze biological duplicate samples obtained from DEG screening, and EBSeq (Leng et al., 2013) was used for non-biological duplicate samples. During the DEG screening, a false discovery rate (FDR) < 0.05 and fold change > 1 were considered standard values. If the DEG fold change was >1, then a FDR < 0.05 was taken to indicate that the DEG was significantly different between the control and test groups.

### Quantitative Real-Time Reverse Transcription PCR Analysis

The transcript levels of DEGs were verified by qRT-PCR. Reverse transcription was accompanied using the PrimeScript^™^ RT Reagent Kit (TaKaRa, Beijing, China). Trans Start^®^ Mix (Trans gen, Beijing, China) was used according to the manufacturer’s instructions to perform qRT-PCR of the genes. The cotton *His3* gene (i.e., *histone 3*) was used as internal control. The relative gene expression levels were calculated with 2^−△△CT^ method (Schmittgen and Livak, 2008).

## Results

### Transcriptome Sequencing and Error Correction

**Figure 1:**
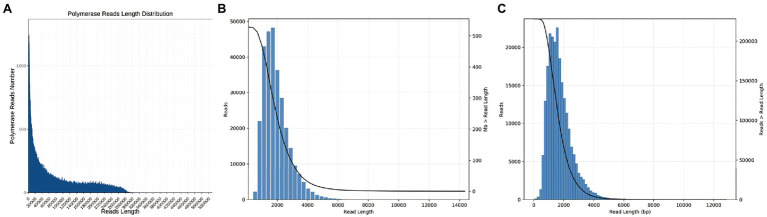
Overall length distribution of the PacBio Sequel data. **(A)** Number and length distribution of polymerase reads. **(B)** Number and length distribution of CCSs. **(C)** Number and length distribution of FLNC reads.

**Figure 2:**
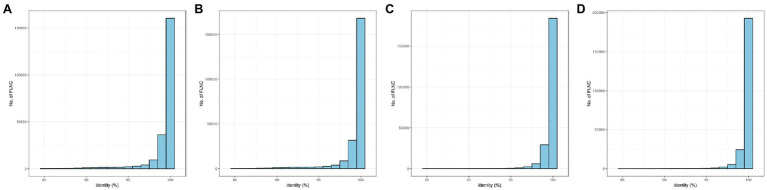
The distribution of PID (percentage-of-identity) before and after error correction. **(A)** Global PID distribution before error correction. **(B)** Local PID distribution before error correction. **(C)** Global PID distribution after error correction. **(D)** Local PID distribution after error correction.

To reduce limited capacity of short-read RNA-Seq in CMS, anther-specific full-length transcriptome analysis of R line was performed in this study. High-quality total mRNAs were pooled from 1–2-, 3-, and 4-mm length flower buds to achieve full-length complete transcripts. The SMRT bell library was constructed and sequenced using the PacBio Sequel platform. After filtering, 337,937 polymerase reads were generated in our sequence. These reads represented more than 25.59 G bases with a mean length of 75,716 bp and N50 length of 162,286 bp (Supplementary Table S1; Figure 1A). After eliminating the adapter from polymerase reads approximately 14,877,534 filtered subreads were obtained, with a mean length of 1,649 bp (Supplementary Table S2). A total of 295,042 circular consensus sequences (CCSs) with an average library depth of 46 passes were produced after subreads integration and error correction by multiple sequencing (Supplementary Table S3). The length distribution of CCSs was consistent with the estimated size of the library (Figure 1B). Then, CCSs were calculated as full-length non-chimeric (FLNC) reads. A total of 228,106 reads were considered to be FLNC with lower artificial concatemers accounted for 77.31% of CCSs. The mean length of FLNC reads was accounting 1728 bp (Figure 1C; Supplementary Table S4). Overall, comprehensively full-length transcripts were achieved to accurately construct splice variants. Illumina HiSeq transcripts were employed to further correct the FLNC reads sequenced by the PacBio Sequel platform. Using LoRDEC software (Salmela and Rivals, 2014), the FLNC reads before and after error correction were compared to the reference genome to measure global and local percentage-of-identity (PID; Figure 2). The PID value was up to 98.49% after error correction (Supplementary Table S5). After adjustment, a total of 221,170 high-qualities FLNC reads were obtained for subsequent investigation (Table 1).

**Table 1:** Classification of reference genome comparison results.

Feature	Precorr	Postcorr	Merge
Unmap	228,106(100.00%)	105(0.05%)	105(0.05%)	
Multiple-best	1,264(0.55%)	1,341(0.59%)	1,178(0.52%)	
Low pid	18,706(8.20%)	6,406(2.81%)	5,653(2.48%)	
High quality map	207,967(91.17%)	220,254(96.56%)	221,170(96.96%)	

### Loci and Isoform Detection and Characterization

**Figure 3:**
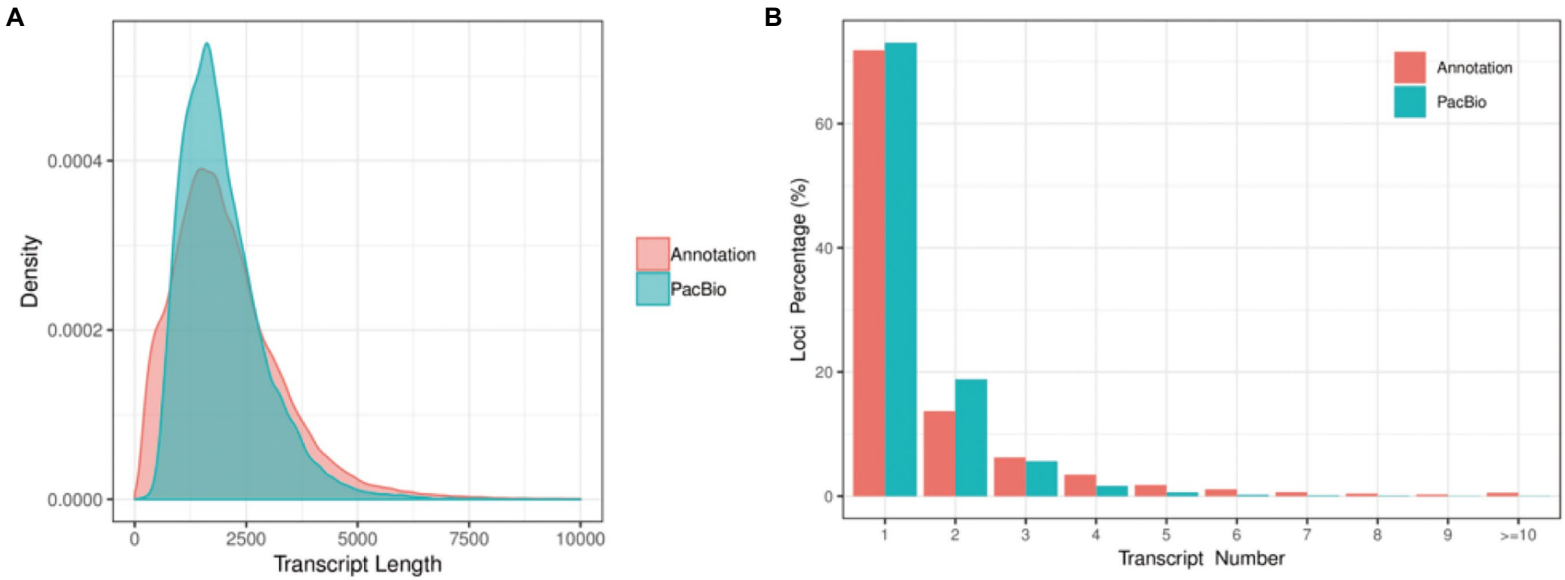
Isoform length density and loci density. **(A)** The length distribution of all isoforms in the PacBio Sequel platform compared to the reference genome. **(B)** The number distribution of isoforms from each locus in the PacBio Sequel platform compared to the reference genome.

Error correction accurately mapped FLNC reads to the reference genome including start site, termination site, and splicing site. This was useful to identify gene loci and isoforms. To calculate length density of isoform, we compared the loci coverage of the PacBio dataset with the *G. hirsutum*_genome_HAU_v1.^6^ In our data, a total of 221,170 error-corrected FLNC reads covered 38,801 isoforms that were allocated to 27,829 loci. About 3,391 loci were 1–2 kb in length, followed by 2–3 kb (7,413), > 3 kb (3,805), and <1 kb (3,391). In the reference genome, about 115,835 isoforms covered 70,199 loci, most loci distributed at <1 kb (18,751), followed by 1–2 kb (26,033), 2–3 kb (15,038), and >3 kb (10,377; Table 2; Figure 3A). Similarly, loci density of isoforms stated that each locus could produce a unique isoform in the reference genome. However, in our data, ~20,321 (73.02%) loci could produce a unique isoform and more than five isoforms covered about 0.35% of the PacBio annotation loci (Figure 3B). Thus, the PacBio dataset provided higher isoform length diversity and loci density than the reference genome which could help to reveal in-depth fertility restorer function of the R line. Almost 80.57% multi-exon isoform and 86.60% multiple-exon FLNC reads contained the same splice donor site at the 5′ end as the reference annotation. These were regarded as full-length transcript implying a relatively higher integrity in structure variant (Table 3).

**Table 2:** Gene structure annotation.

Feature	Annotation.loci.len	PacBio.loci.len
Loci	70,199	27,829
Loci < 1 K	18,751(26.71%)	3,391(12.19%)
Loci 1–2 K	26,033(37.08%)	13,220(47.50%)
Loci 2–3 K	15,038(21.42%)	7,413(26.64%)
Loci ≥ 3 K	10,377(14.78%)	3,805(13.67%)
Total isoform	115,835	38,801

**Table 3:** Evaluation of full-length transcripts in the PacBio data set.

Category	Total	No. of full length	Ratio of full length
Multi-exon isoform	32,079	25,845	80.57%
Multi-exon FLNC	64,956	56,251	86.60%

**Figure 4:**
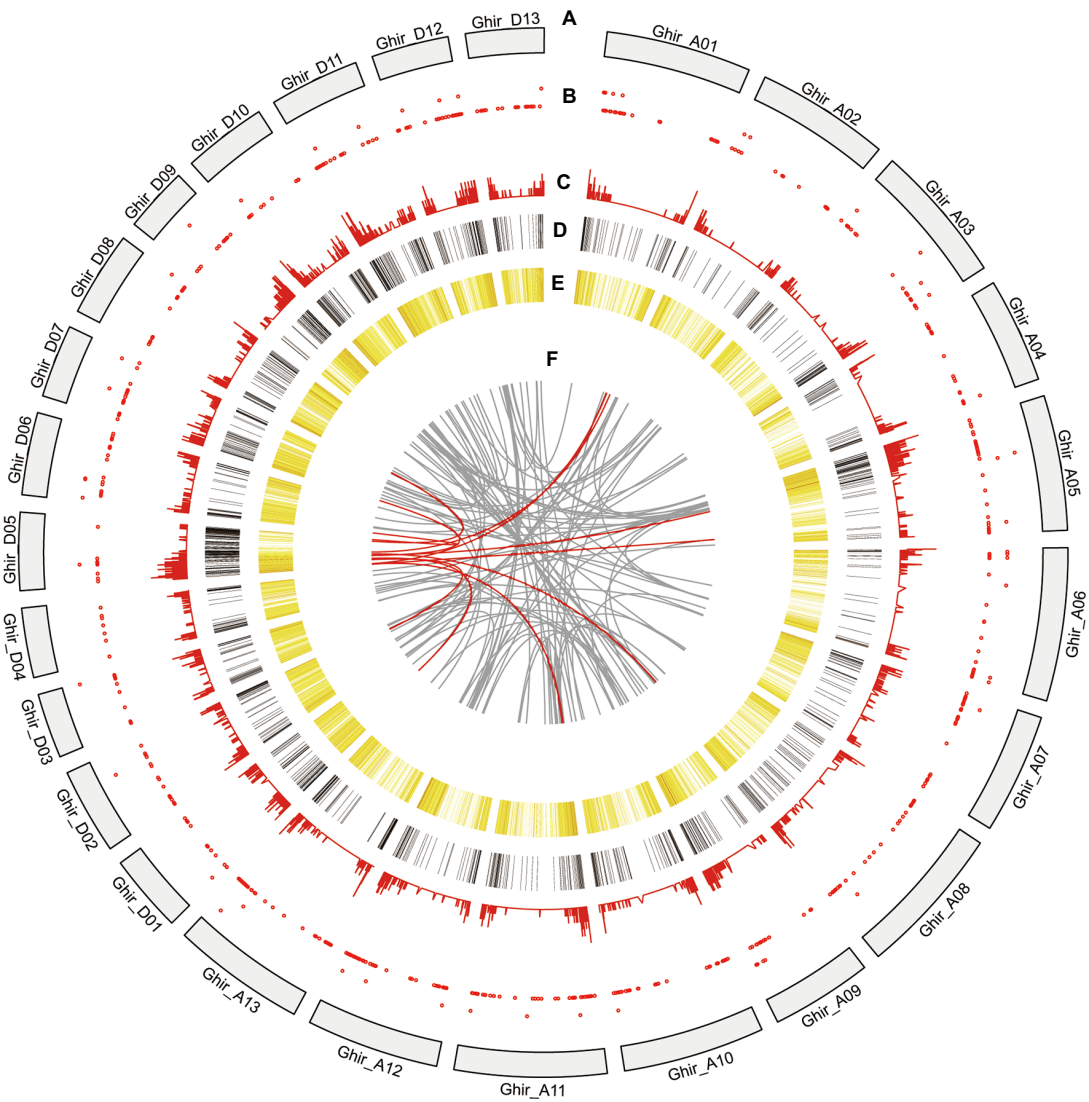
Circos visualization of the PacBio Sequel platform at genome-wide level. **(A)** Twenty-six chromosomes distribution of *G. hirsutum* genome. **(B)** LncRNA density from the PacBio Sequel platform. The closer the red point is to the center, the lower the density. **(C)** APA sites distribution mapped to *G. hirsutum* genome. The closer the line is to the center, the lower the density. **(D)** DEGs of R line compared with A line and B line. The closer the color is to red, the higher the density. Conversely, the closer the color is to black, the lower the density. **(E)** Novel isoforms density from the PacBio Sequel platform. The closer the color is to red, the higher the density. Conversely, the closer the color is to yellow, the lower the density. **(F)** Fusion transcripts distribution. Red line represents fusion transcripts which are involved in the D05 chromosome.

Moreover, the sequenced gene loci and isoforms were compared with the reference annotation to determine novel gene loci or novel isoforms. The published *G. hirsutum* TM-1 genome annotation contains 70,199 loci with 115,835 isoforms. In our PacBio dataset, a total of 38,801 isoforms were identified from 27,829 genes. Of which, 18,010 were known isoforms from known genes. In addition, 2,597 transcripts did not overlap with any annotated genes and were considered as novel genes (Supplementary Table S6). Those novel genes were found to produce 3,174 novel isoforms (Figure 4E). In contrast, 17,617 additional novel isoforms were determined from 12,815 known genes. Out of 3,174 novel isoforms, 1,313 (41.37%) were single-exon isoforms, and 1861 (58.63%) were multiple-exon isoforms. Furthermore, among 16,857 known loci novel isoform, there were 1789 (10.61%) single exon isoform and 15,068 (89.39%) multiple exon isoforms. The greater number of identified novel genes and isoforms were useful for reliable genes annotation within the candidate interval of *Rf_2_*.

### Functional Annotation of Novel Isoforms

**Figure 5:**
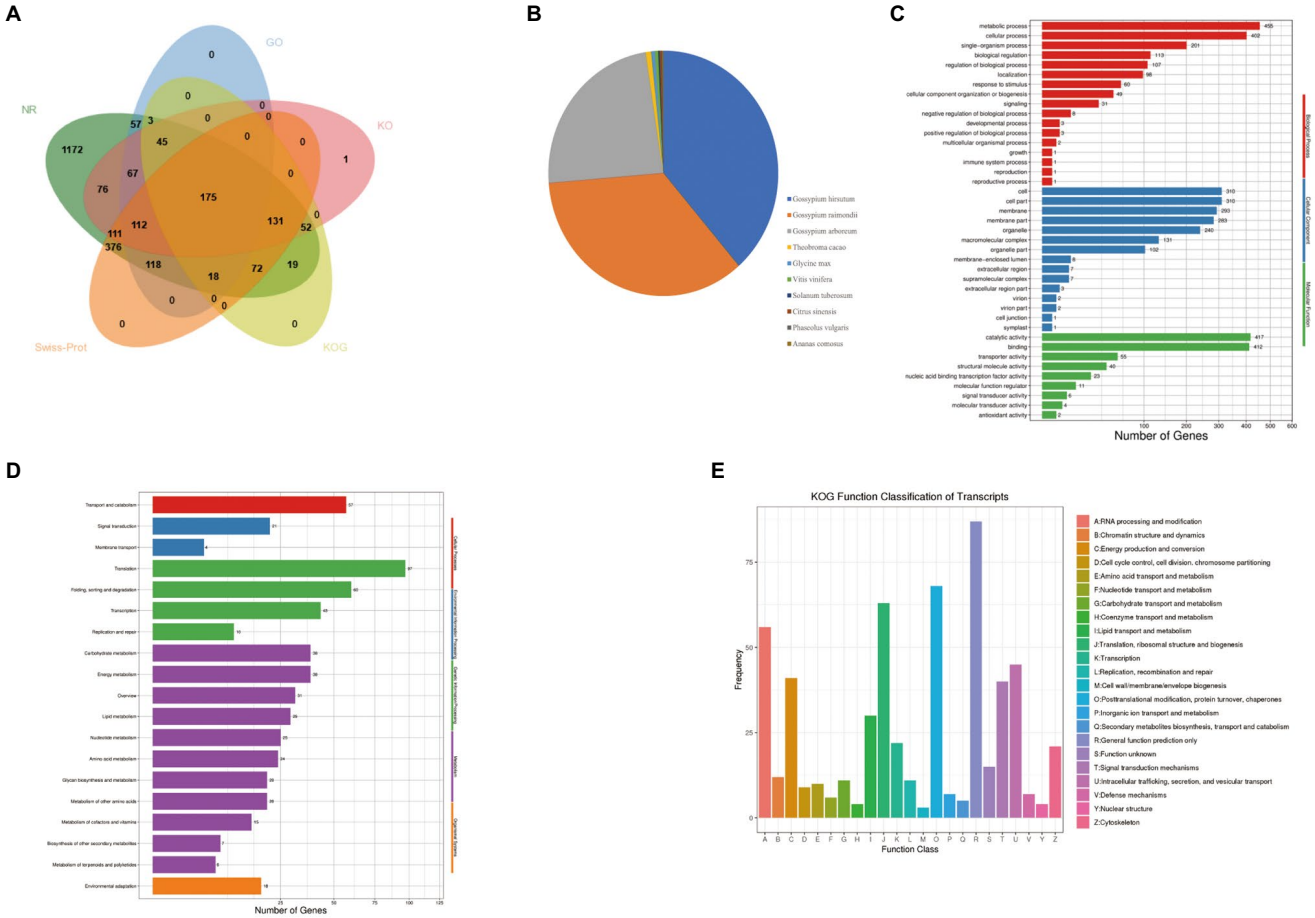
Function annotations of novel isoforms identified by the PacBio Sequel platform. **(A)** The number statistics of novel isoforms in NR GO, KEGG, KOG databases. **(B)** Distribution of novel isoforms in Nr homologous top 10 species. **(C)** Distribution of novel isoforms in GO terms. **(D)** Distribution of novel isoforms in KEGG pathway. **(E)** Distribution of novel isoforms in KOG.

In this study, all 3,174 novel isoforms functional annotation were retrieved from different databases. It was observed that 82.04% isoforms were annotated on NCBI non-redundant protein sequences (NR), 26.84 on Gene Ontology (GO), 24.26% on EuKaryotic Orthologous Groups (KO), 16.23% on eukaryotic Ortholog Group (KOG), and 35.07% on Swiss-Prot Protein Sequence (Swiss-Prot) database However, a total of 377 (17.93%) novel isoforms were unannotated (Supplementary Table S7). A total of 175 novel isoforms had significant hits in all five databases (Figure 5A). In the NR database, the largest three groups of novel isoforms were distributed in *G. hirsutum* (969), *G. raimondii* (880), and *G. arboreum* (599; Figure 5B). GO analysis assigned the enrichment of 852 isoforms to various biological process, cellular component, and molecular function. It was found that 863 GO terms were enriched in biological process. Out of which, metabolic (53.40%), cellular (47.18%), and single-organism (23.59%) process had major proportion. In addition, many of the terms in biological process were related with energy metabolism such as ATP metabolic process, ATP synthesis coupled electron transport, oxidation–reduction process, and energy derivation by oxidation of organic compounds. A total of 257 GO terms were detected in category of cellular component. The cell (36.38%), cell part (36.38%), and membrane (34.39%) were the largest three enrichment terms. For a particular interest, six novel isoforms were enriched functions into mitochondrial proton-transporting ATP synthase complex, and catalytic core *F*(1). Our data showed that 442 GO terms were assigned to molecular function and the most highly abundant terms were catalytic activity (48.94%) and binding (48.63%; Figure 5C). To identify the enrichment pathways, a total of 695 novel isoforms were subjected to 98 KEGG pathways. Novel isoforms in KEGG pathways were consisted of five hierarchies, e.g., cellular processes, environmental information processing, genetic information processing, metabolism, and organismal systems. Among these terms, the most abundant hierarchy was metabolism (366, 52.66%) followed by genetic information processing (236, 33.96%; Figure 5D). KOG analysis had shown that 515 novel isoforms were assigned to 23 categories and the largest three classes were general functional prediction only (87, 16.90%), posttranslational modification, protein turnover, chaperones (68, 13.20%), and Translation, ribosomal structure and biogenesis (63, 12.23%; Figure 5E).

### LncRNA and ORF Prediction of Novel Isoforms

LncRNAs perform regulatory functions and play critical role for post-transcription, transcription, and epigenetic changes (Huang et al., 2018). A total of 652 novel isoforms were predicted to be lncRNAs with a mean length of 1,029 bp which counted for 3.25% of all novel isoforms (Supplementary Table S8). About 215 lncRNAs (32.98%) were longer than 1,000 bp and 9 lncRNAs were longer than 3,000 bp. Mapping of the predicted lncRNAs to *G. hirsutum* 26 chromosomes was presented using Circos visualization software (Figure 4A). It revealed that 652 lncRNAs were randomly distributed (Figure 4B). Moreover, open reading frames (ORFs) were predicted with transDecoder software. It resulted that a total of 16,390 novel isoforms were predicted with ORF. Then, the density and length distributions of coding sequences (CDS) were investigated. The encoded peptide sequences are listed in Supplementary Table S9.

### AS and APA Identification

**Figure 6:**
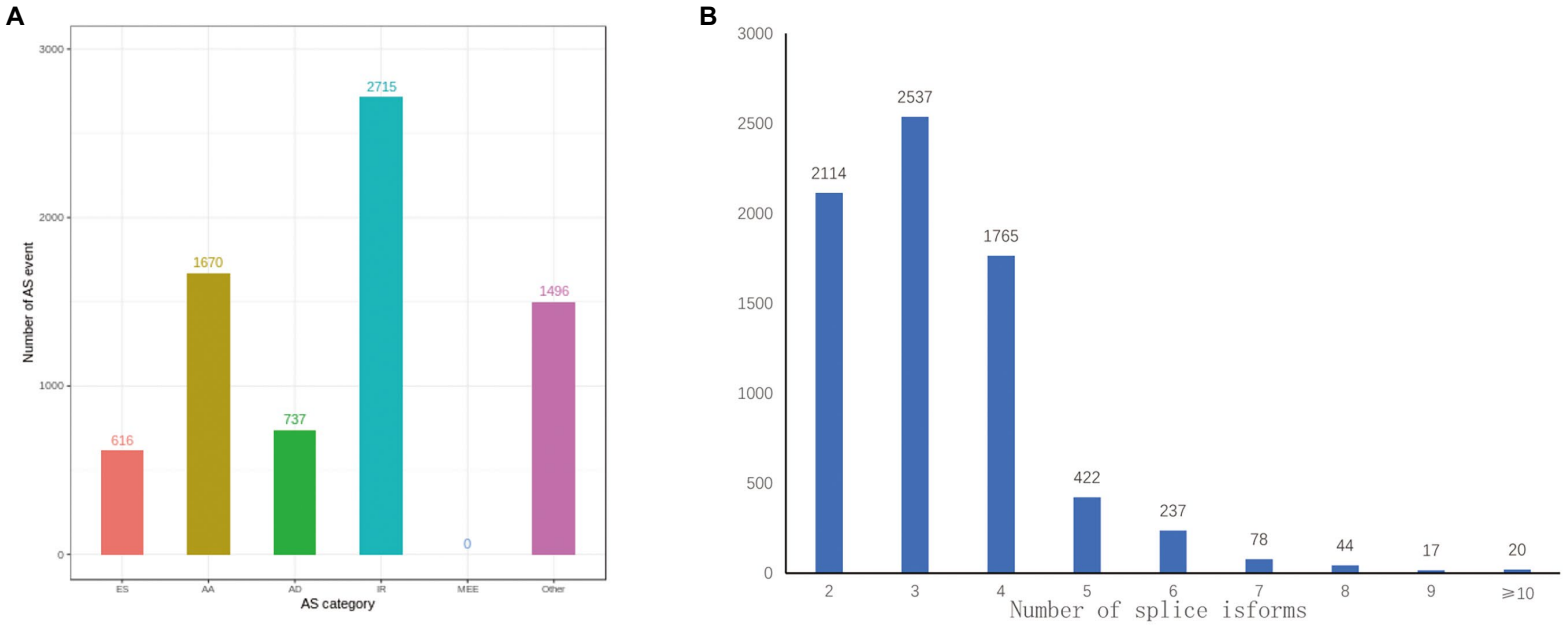
Identification of AS events. **(A)** The distribution of AS events in loci detected by the PacBio Sequel platform. **(B)** Distribution of loci that produce two or more splice isoforms detected by the PacBio Sequel platform.

AS increased the complexity of transcriptomes and proteomes in accordance to the diverse splice modes rather than by amplifying the number of genes in cells or tissues (Wang et al., 2008). A total of 27,829 loci corresponding to 38,801 isoforms underwent 7,234 AS events *via* different spliced modes. It included 616 ES events, 1,670 AA events, 737 AD events, 2,715 IR events, and 1,496 other AS events. These detections stated that the distribution of AS events was much higher in anthers of R line (Figure 6A). Moreover, two or more isoforms were found in 7,234 gene loci in our PacBio Sequel platform analysis. Ten or more splice isoforms were detected in 20 genes (Figure 6B).

**Figure 7:**
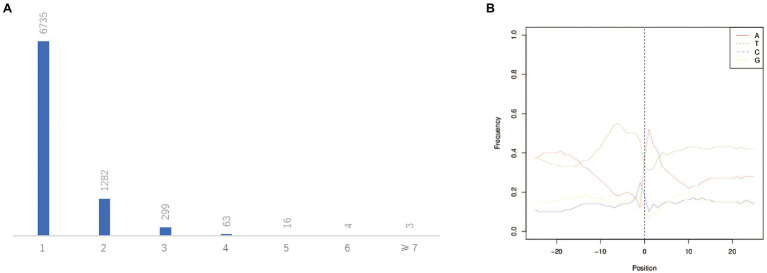
APA analysis predicted by the PacBio Sequel platform. **(A)** The distribution of poly-A sites per gene. **(B)** Nucleotide distribution around poly-A cleavage sites.

The post-transcriptional modification process of pre mRNA to mature mRNA mainly includes the addition of a 7-methylguanosine cap at the 5′-end, intron splicing, and 3′-end formation by cleavage and polyadenylation (de Almeida et al., 2010). The specific position of the poly-A tail at the 3′-end is variable and this variation may affect the binding of microRNA or RNA-binding protein to mRNA as well as the process of RNA splicing and translation. By investigating the 3′-end of transcripts a total of 10,580 poly-A sites were detected from 8,402 genes. Of which, 1,667 genes showed alternative polyadenylation (APA; Figure 4C). A total of 6,735 genes had at least one poly-A site while 23 genes had more than four poly-A sites (Figure 7A). The largest number of poly-A sites was 10 that were found in *Ghir_D06G000070* and *Ghir_D11G012090*. Then, nucleotide distribution of the 30 nts in upstream and downstream of all poly-A sites were analyzed. Consistent with other species, the poly-A sites from our PacBio dataset showed a nucleotide bias with an enrichment of uracil (U) upstream and adenine (A) downstream (Figure 7B).

### Fusion Transcript Identification

A fusion transcript refers to a new gene formed by splicing together two or more separate genes which are known as chimeric transcripts. The genomic structural variation, transposition, or trans-splicing after transcription caused generation of fusion transcripts. A total of 114 fusion transcripts related to 225 annotated genes were identified in this study (Supplementary Table S10). According to the chromosomal distribution, 109 inter-chromosome and 5 intra-chromosome fusion transcripts were detected. Previous studies have indicated that most fusion transcripts are composed of two genes (Weirather et al., 2015). Likewise, all the 114 fusion transcripts were composed of two genes in our data. Intriguingly, 11 fusion transcripts were convoluted on the D05 chromosome (Figure 4F). Specifically, *m64033_190821_201011/87884027/ccs* is composed of *Ghir_A01G016800.1* (Niemann-Pick C1 protein) and *Ghir_D05G004620.1* (Pollen-specific protein SF21) genes and experimentally validated in anthers of CMS-D8 system using RT-PCR. Thus, the *m64033_190821_201011/87884027/ccs* is certainly in the anthers transcription (Supplementary Figure S1).

### Analysis of Unmapped FLNC Reads and DEGs

**Figure 8:**
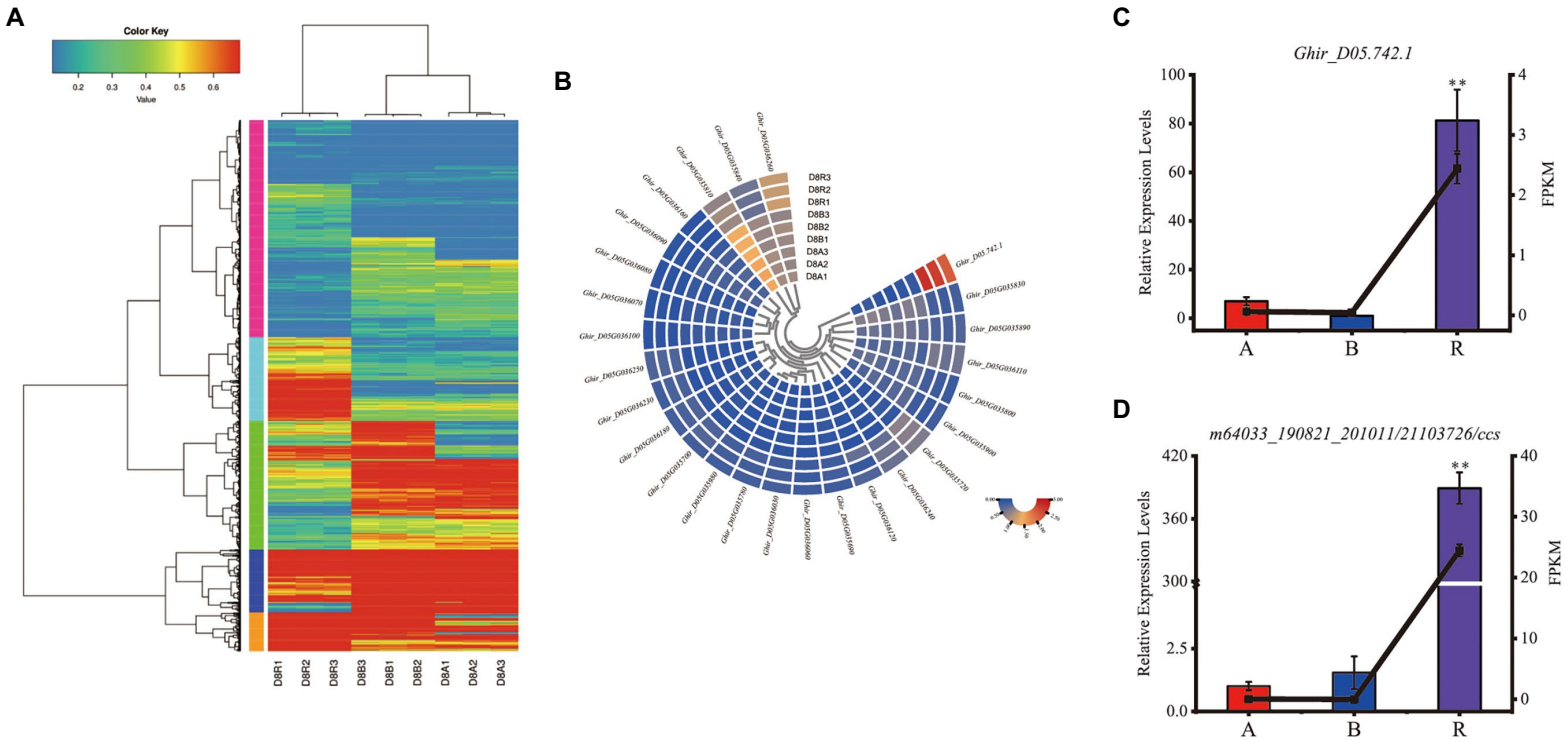
The expression of DEGs. **(A)** Heat map of DEGs in R line compared with A line and B line. **(B)** Heat map of FPKM values of differentially expressed genes in candidate interval. A1, A2, A3: sterile line, B1, B2, B3: maintainer line, and R1, R2, R3: restorer line. **(C)** Expression patterns of *Ghir_D05.742.1*. **(D)** Expression patterns of *m64033_190821_201011/21103726/ccs*. Histograms indicate relative expression levels. Lines indicate FPKM. (^**^p < 0.01) The asterisks indicate that the difference in gene expression in the A, B, and R lines was highly significant.

Since, *Rf_2_* is derived from of *G*. *trilobum* (Stewart et al., 1992; Zhang and Stewart, 2001a), the sequence of *Rf_2_* might be not available in the reference genome of *G. hirsutum*. The unmapped reads were further analyzed and 76 isoforms were identified after de-redundancy with ci-hit software, resulting in identification of 10 isoforms which were expressed in the R line with FPKM > 1. As compared with the CMS-D8 A line, the genes that met the default criteria with FDR < 0.05, log FC > 1 or log FC < −1 by DEseq2 software were assigned as DEGs. The 6,084 differentially expressed isoforms from 4,112 DEGs were discovered. Among the differentially expressed isoforms, 3,187 isoforms were up-regulated and 2,897 isoforms were down-regulated (Supplementary Table S11). In DEGs, 2,211 were up-regulated 1,901 were down-regulated. The data also showed that 520 of the novel isoforms were up-regulated and 232 were down-regulated. Comparative analysis showed that 1,456 DEGs (Figure 4D), 161 novel isoforms, and 56 novel genes were upregulated in R line then A line and B line (Figure 8A). Subsequently, novel isoforms specifically expressed in R line with log FC > 3 or FPKM > 1 value and FPKM < 1 in A line and B line were identified. As a result, 39 novel isoforms specifically expressed in R line were screened out. These R line up-regulated genes especially R line-specific genes may co-respond to the expression of restorer gene *Rf_2_* and most likely regulate fertility restoration function in CMS-D8 cotton.

### Identification the Genes Involved in Fertility Restoration

According to the previous study, *Rf_2_* is located within the candidate interval of 1.48 Mb on the D05 chromosome (Feng et al., 2021). Hence, the genes in the candidate interval were analyzed in this study. It was determined that 25 of 76 genes were differentially expressed in CMS-D8 system and four novel isoforms were identified including *Ghir_D05.742.1* which was specifically expressed (Figure 8B). Considering that the *Rf_2_* gene is a foreign gene introduced into upland cotton, the analysis of unmapped reads shows that one isoform named *m64033_190821_201011/21103726/ccs* was specifically expressed in R line. The *Ghir_D05.742.1* was annotated as disease resistance protein At4g27190-like in NR database of NCBI, but the *m64033_190821_201011/21103726/ccs* could not be annotated. The qRT-PCR showed that the relative expression levels of *Ghir_D05.742.1* and *m64033_190821_201011/21103726/ccs* in the restorer line was significantly higher than A and B lines (Figures 8C,D). These results suggested that *Ghir_D05.742.1* and *m64033_190821_201011/21103726/ccs* are closely related to *Rf_2_* gene or these two genes are candidate genes of *Rf_2_*.

## Discussion

Hybrid breeding has potential to reduce recent the decline in upland cotton production in the world. CMS is a common phenomenon used to produce hybrid seeds in flowering plants. The CMS system restore fertile pollen as a result of the interaction between the mitochondrial and nuclear genomes (Touzet and Meyer, 2014). CMS is required for efficient commercial hybrid breeding of various agronomic crops. Among various techniques of hybrid seed production, the CMS system has been proven to be an ideal and economically effective tool than convention hybrid seed production. The CMS-D8 system in upland cotton with cytoplasm introduced from *G. trilobum* is one of the main CMS systems in cotton with stable inheritance and complete male abortion. The restorer line of CMS-D8 system contains the restorer *Rf_2_* gene introgressed from *G*. *trilobum*. Although, the *G. hirsutum* genome sequence has been released by cotton researcher community, there are few reports on the full-length transcriptome of cotton, its genome and transcriptome information still need to be further explored. At present, the research of transcript paired-end sequence has extensively been performed in CMS cotton. Wu et al. (2017) discovered 1,464 DEGs by genome-wide comparative transcriptome analysis of CMS-D2 and its maintainer and restorer lines; Yang et al. (2018) discussed the relationship of CMS and its ability to eliminate ROS using the transcriptome of CMS and maintainer line of CMS-D8 cotton. These studies only provided expression changes of transcripts but not reported the novel isoforms and novel genes in cotton CMS systems. Li et al. (2021) recently published the full-length transcripts for restorer line of CMS-D2 system which provided all full-length transcripts with many new isoforms and gens for R line. Since, CMS-D2 aborts in sporophyte and CMS-D8 is gametophyte abortion, the *Rf_1_* of CMS-D2 system was from *G. harknessii*, and *Rf_2_* of CMS-D8 system source from *G*. *trilobum*, and restoration function mechanism may be different. There have already been no reports about the full-length transcriptome of the CMS-D8 restorer line. So, we highly needed the full-length transcriptome to accelerate the research of restoration function of the restorer line in CMS-D8 system. In our study, Iso-seq analysis was performed on the mixed anther, full-length transcripts were identified from 70,199 loci, novel isoform, and fusion transcript, and candidate genes were recognized to facilitate the research on fertility restoration function in R line.

With advance in sequence technology as well as breakthrough in bioinformatics has revealed genetic control of complex trait. Second generation sequencing made vast improvement compared to Sanger sequencing. However, short reads certainly caused assembly gaps and inaccurate detection of isoforms, AS events, and APA sites. In recent years, Pacbio full-length transcriptome sequencing offers an alternative approach to accurately identify AS, APA, and fusion transcripts for target traits (Huang et al., 2022; Tong et al., 2022). Our results identified 7,234 AS events, 1,667 APA, and 114 fusion transcripts that exposed the complexity of transcripts in R line. The functional verification of fusion transcripts has been carried out in some studies in human diseases (Agostini et al., 2018; Siegfried et al., 2019), but rarely reported in plants. This study verified the existence of the fusion transcript, *m64033_190821_201011/87884027/cc* but unable to study function of this fusion transcript. The *m64033_190821_201011/87884027/ccs* contains partial sequences of *Ghir_A01G016800.1* (Niemann-Pick C1 protein) and *Ghir_D05G004620.1* (Pollen-specific protein SF21). It may have the function similar to *Ghir_D05G004620.1* which probably regulates anthers development.

Previous studies reported that the restoration of function conditioned by one dominant restorer *Rf_2_* gene in R line of CMS-D8 cotton (Zhang and Stewart, 2001a). At the present, there is no report about the gene expression profiles affected by the *Rf_2_* in R line. Our study analyzed 39 novel genes specifically expressed in R line. The result of qRT-PCR showed that the relative expression levels of *Ghir_D05.742.1* and *m64033_190821_201011/21103726/ccs* in the restorer line were significantly higher than A and B lines. This result revealed specific gene expression of R lines compared with upland cotton (TM-1). Our results provide an important foundation for further studies which aims to explore molecular mechanism of the interactions between the *Rf_2_* and the CMS-D8 cytoplasm.

## Conclusion

The Iso-seq developed by Pacific Bio-sciences generates full-length transcripts without assembly. The analyzed full-length transcriptome of R line of CMS-D8 cotton yielded a total of 295,042 CCSs. Of these, 228,106 transcripts were identified as FLNCs. Meanwhile, 3,174 novel isoforms from 2,597 novel gene loci, 652 lncRNAs predicted from novel isoforms, 7,234 AS, 114 fusion transcripts, and 1,667 APA were identified. Further DEGs comparative analysis stated that 161 novel isoforms and 56 novel genes were up-regulated in R line. In particular, 39 novel isoforms specifically showed expression changes in R line. Finally, our study found two key genes named as *Ghir_D05.742.1* and *m64033_190821_201011/21103726/ccs* closely related to restoration function in restorer line of CMS-D8 system. These results provided new insights into novel isoforms, AS events, and candidate gene discovery of R line. Additionally, our datasets will offer a platform to explore in-depth fertility restoration mechanism of R line in CMS-D8 cotton.

## Data Availability Statement

The datasets presented in this study can be found in online repositories. The names of the repository/repositories and accession number(s) can be found at: NCBI—PRJNA685585, SAMN28725462, SAMN17088084, and SAMN17088083.

## Author Contributions

JW, CX, and ZL designed the experiments. LG, TQ, HT, XQ, and HW did the field management. JF, YL, MZ, and XZ performed data analysis and qRT-PCR. JF, JW, JZ, and KS contributed to the preparation of the manuscript. All authors contributed to the article and approved the submitted version.

## Funding

This work was sponsored by funds from the Zhongyuan Academician Foundation (212101510001), the Agricultural Science, Technology Innovation Program of Chinese Academy of Agricultural Sciences and the Fundamental Research Funds for State Key Laboratory of Cotton Biology (CB2022C05).

## Conflict of Interest

The authors declare that the research was conducted in the absence of any commercial or financial relationships that could be construed as a potential conflict of interest.

## Publisher’s Note

All claims expressed in this article are solely those of the authors and do not necessarily represent those of their affiliated organizations, or those of the publisher, the editors and the reviewers. Any product that may be evaluated in this article, or claim that may be made by its manufacturer, is not guaranteed or endorsed by the publisher.

## Footnotes

^1^ http://www.pacb.com/products-and-services/analytical-software/smrt-analysis/

^2^ http://www.bioinformatics.babraham.ac.uk/projects/fastqc

^3^ https://github.com/juliangehring/GMAP-GSNAP

^4^ http://lilab.research.bcm.edu/cpat/

^5^ http://transdecoder.sf.net

^6^ https://www.cottongen.org/species/Gossypium_hirsutum/HAU-AD1_genome_v1.0_v1.1

## Supplementary Material

Supplementary Figure S1 The analysis of m64033_190821_201011/87884027/ccs.
