# Supplementary figures and images for: Transcript Complexity and New Insights of Restorer Line in CMS-D8 Cotton Through Full-Length Transcriptomic Analysis
Source: Front Plant Sci. 2022 Jun 21;13:930131. doi: 10.3389/fpls.2022.930131 (PMC9253813; doi:10.3389/fpls.2022.930131)

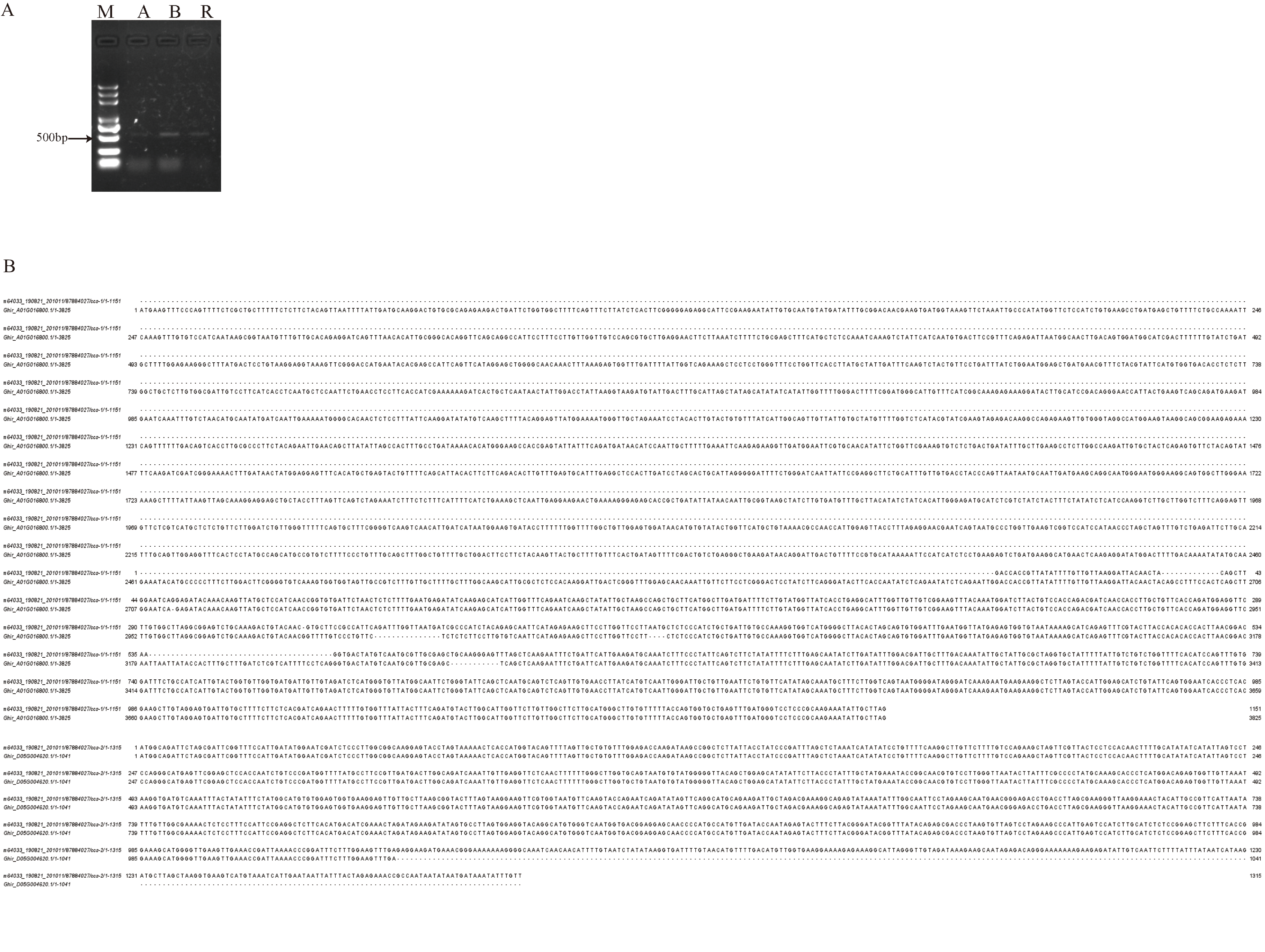

Supplement: Supplementary Figure S1 — The analysis of m64033_190821_201011/87884027/ccs. [file Image_1.PNG]
